# Supplementary figures and images for: Ciliary Motility Decreased by a CO2/HCO3−-Free Solution in Ciliated Human Nasal Epithelial Cells Having a pH Elevated by Carbonic Anhydrase IV
Source: Int J Mol Sci. 2024 Aug 21;25(16):9069. doi: 10.3390/ijms25169069 (PMC11354224; doi:10.3390/ijms25169069)

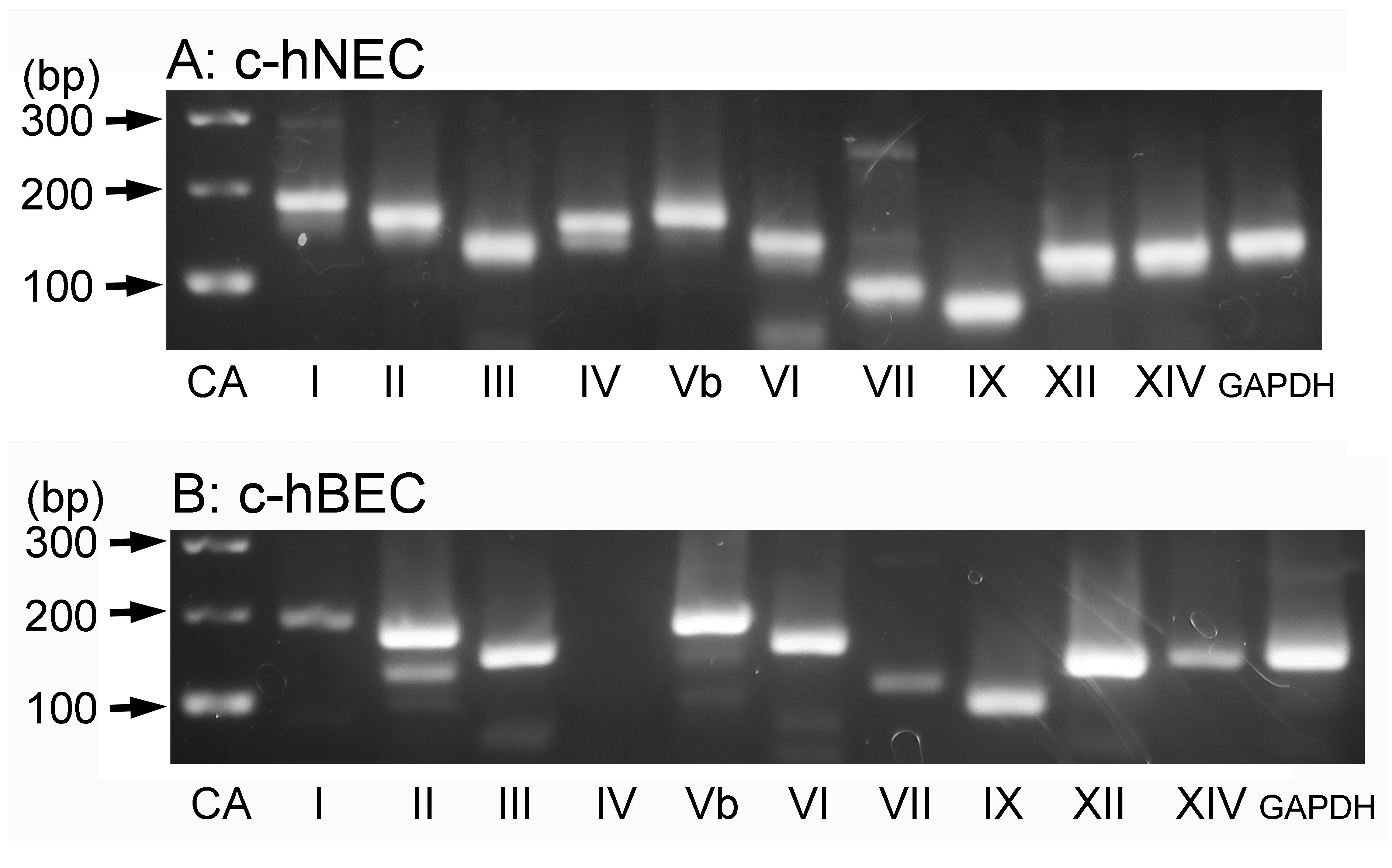

Supplement: Supplementary file 1 [file ijms-25-09069-s001.zip › Figure S1.jpg]

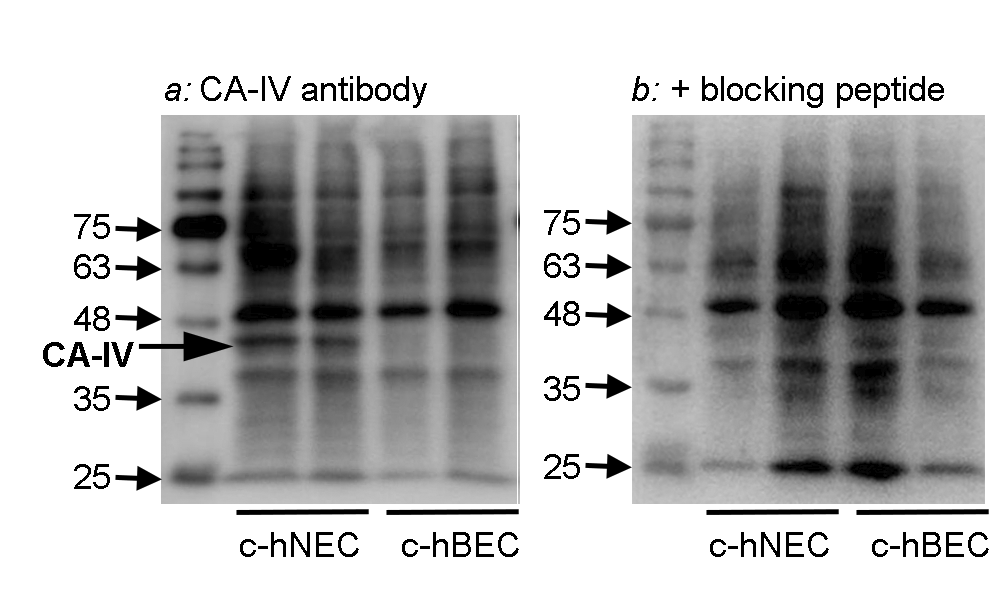

Supplement: Supplementary file 1 [file ijms-25-09069-s001.zip › Figure S2.tif]
